# Dry needling for orofacial pain: a systematic review and meta-analysis of randomized clinical trials

**DOI:** 10.1097/PR9.0000000000001208

**Published:** 2024-11-20

**Authors:** Adrian Kuzdzal, Edzard Ernst, Pawel Posadzki, Zbigniew Wronski

**Affiliations:** a College of Medical Sciences, Institute of Health Sciences, University of Rzeszów, Rzeszów, Poland; b Complementary Medicine, Peninsula Medical School, University of Exeter, Devon, Exeter, United Kingdom; c Faculty of Rehabilitation, University of Physical Education, Cracow, Poland; d Department of Physiotherapy Fundamentals, Faculty of Medicine and Dentistry, Medical University of Warsaw, Warsaw, Poland

**Keywords:** Dry needling, Orofacial pain, Temporomandibular disorders, Effectiveness, Safety, Systematic review, Meta-analysis

## Abstract

Orofacial pain (OFP) is an ache in the front part of the head and typically has musculoskeletal, dental, neural, or sinogenic origin. Dry needling (DN) is the insertion of a solid sterile fine needle through the skin aimed at deactivating the myofascial trigger points has extensively been studied in randomized clinical trials (RCTs). This systematic review was aimed at evaluating the evidence base for the effectiveness of DN for OFP. We searched Medline, Cochrane Central, and Web of Science (from their respective inceptions to February 2024) for RCTs evaluating the effectiveness of DN in patients with OFP. Studies with individuals suffering from cervicogenic or tension type headaches as well as observational studies were excluded. Primary outcomes were pain intensity and severity; secondary outcomes were disability, quality of life, and adverse effects. The review adhered to the methods described by in the Cochrane Handbook. Twenty-four RCTs with a total of 1,318 patients suffering from OFP were included. Most had an unclear or high risk of bias, and the quality of the evidence ranged from very low to low for all comparisons and outcomes. A meta-analysis suggested that, compared with usual care alone, DN + usual care had no effect on pain intensity (visual analogue scale) (standardized mean difference = −1.89, 95% confidence intervals −5.81 to 2.02, very low certainty evidence) at follow-ups of up to 6 weeks. There is little reliable evidence for the effectiveness of DN in reducing OFP. Future, larger, more rigorous studies might reduce the existing uncertainties.

## Abstract 2

Supplemental Digital Content is Available in the Text.

After evaluating the available literature, this systematic review found disappointingly little compelling evidence for the effectiveness of dry needling in reducing orofacial pain.

## 1. Introduction

Orofacial pain (OFP) can be defined as an ache in the front part of the head with its origin below the orbitomeatal line, above the neck, and anterior to the ears, including pain within the mouth.^5,8,40,49^ Orofacial pain is typically caused by temporomandibular disorders, trigeminal neuralgia, or atypical facial pain, with approximately 42% of diagnoses corresponding to myofascial pain or myofascial trigger points.^33^ The prevalence of OFP varies considerably from 5% to 57% across the studies depending on sociodemographics, geographical regions, underlying conditions, and diagnostic criteria used.^31^ Orofacial pain may lead to functional limitations, anxiety, depression, sleep disturbances, and poor quality of life.^7,8^ It causes considerable suffering and imposes substantial costs to health care systems.^18,25^ Today, there is only limited knowledge about aetiology and pathophysiology of the condition, and clinicians are uncertain as to what the optimal treatments might be.^10^ With its intricate interplay of neuroanatomical and neurophysiological networks (nerves, muscles, joints, tendons, fascia) psychological and socioenvironmental factors, OFP requires a comprehensive understanding to address its underlying mechanisms and to develop effective treatment strategies.

Dry needling (DN) refers to the insertion of monofilament stainless steel needles into muscles, ligaments, tendons, connective tissue, scar tissue, and perineural tissue for the management of neuromusculoskeletal conditions.^17,34^ The technique that is not dissimilar to acupuncture have been shown to elicit biochemical, biomechanical, endocrinological, and neurovascular changes associated with reductions in pain and disability.^19,30^ Those analgetic effects may include a release of endorphins, serotonin, norepinephrine or activate large beta fibres, thereby inhibiting the transmission of pain signals through smaller A-delta and C fibres at the spinal level.^9^

The rationale for conducting this systematic review (SR) is at least 2-fold. First, dozens of trials evaluating DN as a sole therapy or in conjunction with, for example, manual therapies have been published in recent decades. Those trials have, however, generated conflicting and contradictory results.^7,15–17,20^ Second, a few reviews (some narrative and thus prone to bias) focussing on DN have generated positive conclusions, implying biological plausibility of the technique and its applicability in clinical practice. Therefore, in an attempt to clear up the confusion, this SR is aimed at critically evaluating the available evidence for or against the effectiveness of DN for OFP.

## 2. Methods

### 2.1. Search strategy and data sources

Searches were performed in the following electronic databases: Medline, Cochrane Central, and Web of Science (from their respective inceptions to February 2024) using the search terms constructed over 2 concepts: DN and OFP (with methodological filter, please see the Appendix 1, available at http://links.lww.com/PR9/A260 for full details). In addition, bibliographies of all thus identified studies were scanned for any relevant papers. We considered studies, both published and unpublished, in any language.

### 2.2. Data selection, extraction, and management

The search results from those databases were combined in a single EndNote (20.1) library, and duplicate records were removed. Titles and abstracts identified through the electronic database searching were screened by 1 reviewer (P.P.) and validated by another (A.K.). During that first screening stage, any references that did not meet the inclusion criteria of the review were excluded (no reasons were provided). Subsequently, full papers were retrieved for all the potentially relevant papers and were examined in detail to determine whether they meet the criteria for inclusion by those same reviewers. With respect to both screening stages, any discrepant views were resolved through a consensus. The selection of studies is reported in accordance with the recent Preferred Reporting Items for Systematic Reviews and Meta-Analyses 2020 guidelines.^39^ Using a custom-made abstraction form (Microsoft Excel), we extracted any details of the study design, populations, treatments, comparators, outcome measures, and quantitative results (effectiveness of the interventions). Two independent reviewers performed data extractions (A.K., P.P.) with a third reviewer acting as arbiter (Z.W.). Any disagreements were settled through a consensus. Wherever feasible, we attempted to obtain missing data from the original study's authors. In case of any missing data, for example, standard deviation, we calculated them from other statistics, such as 95% confidence intervals (CIs), standard errors, or *P* values using RevMan 5.4 calculator and available formulae.^24^

### 2.3. Eligibility criteria

We included all randomized clinical trials (RCTs), testing the effectiveness of DN in OFP (regardless of the classification/criteria used) in human subjects (see Supplementary Table 1, available at http://links.lww.com/PR9/A260). Any type of controls and outcome measures were permissible. We excluded participants suffering from cervicogenic or tension type headaches, as well as observational studies as they are considered lower than RCTs level of evidence. Primary outcomes consisted of pain intensity and severity; secondary endpoints were disability, quality of life, and adverse effects (AEs) at follow-ups of up to 12 months.

### 2.4. Risk of bias assessment

We used the Cochrane Risk of Bias tool to assess the methodological risk of bias of all reviewed studies by 2 independent reviewers. The Cochrane Handbook for Systematic Reviews of Interventions recommends explicitly reporting the following individual elements for RCTs: random sequence generation; allocation concealment; blinding (participants, personnel); blinding (outcome assessment); completeness of outcome data (attrition bias), selective outcome reporting (relevant outcomes reported); and other sources of bias (baseline imbalances).^23^

### 2.5. Data synthesis

Where studies were homogeneous enough in populations, interventions, comparators, outcomes, and study designs, we pooled them quantitatively in a meta-analysis. For studies assessing the same continuous outcomes, we estimated standardized mean differences (SMDs) (for different scales) between groups, along with 95% CIs. For dichotomous outcomes, risk ratios were estimated along with 95% CIs. The results of meta-analyses were displayed in forest plots, which provide effect estimates and 95% CIs for each individual study as well as a pooled effect estimates and 95% CI. We only combined results of studies that reported uniform and comparable timing of outcome assessment. All meta-analyses were performed using RevMan 5.4 (desktop version) and adhering to the statistical guidelines described in the Cochrane Handbook.^23^ We used Generic Inverse Variance method for trials reporting change scores. A random-effects model was chosen as it provides a more conservative estimate of effect. We assessed heterogeneity through a visual inspection of the overlap of forest plots and by calculating the χ^2^, τ^2^ tests, and *I*^2^ inconsistency statistics.

### 2.6. Analysis issues

When a study had more than 1 active treatment arm, we labelled the study arms as “a,” “b,” “c,” and so forth. If more than 1 intervention arm was relevant for a single comparison, we compared the relevant DN arm with the least active control arm to avoid double-counting of data. Where several endpoints were available, we choose the longest follow-up for any between group differences.

### 2.7. Subgroup and sensitivity analyses

We planned to perform subgroup analyses by the depth and type of needling, intensity, and frequency of the intervention. However, they were deemed implausible given the small number of available studies.

### 2.8. Quality/certainty of the evidence (Grading of Recommendations Assessment, Development, and Evaluation)

We prepared “Summary of findings” tables to present the results for the main primary outcomes, based on meta-analysis or/and narrative synthesis. We converted results into absolute effects when possible and provided a source and rationale for each assumed risk cited in the table(s). Two authors independently (A.K., P.P.) assessed the overall quality of the evidence as implemented and described in the chapter 14 of the Cochrane Handbook for Systematic Reviews of Interventions.^41^ We considered the following criteria to assess the certainty of evidence: limitations of studies (risk of bias), inconsistency, indirectness, imprecision and publication bias, and downgrade the quality where appropriate for primary and secondary outcomes.

The protocol for this SR has been registered with PROSPERO: CRD42024505908.

## 3. Results

### 3.1. Study characteristics

**Figure 1.**
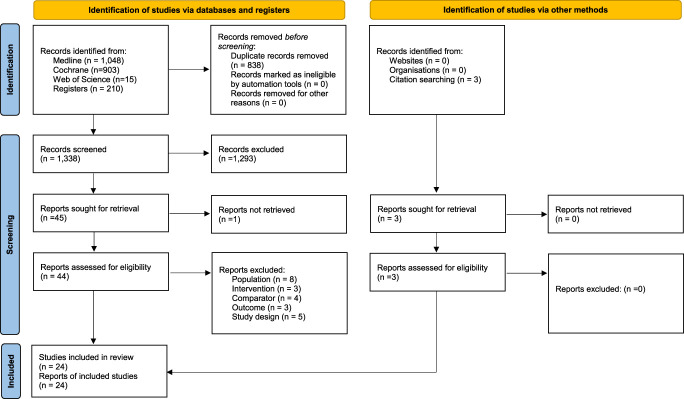
Preferred Reporting Items for Systematic Reviews and Meta-Analyses flow diagram.

**Figure 2.**
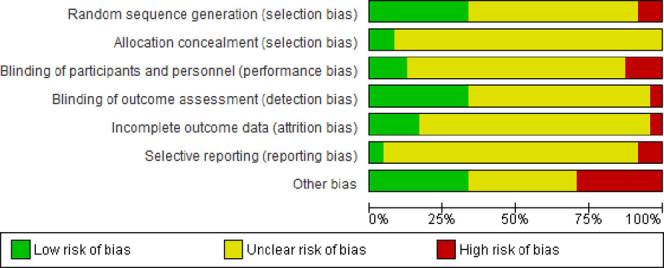
Risk of bias graph: review authors' judgements about each risk of bias item presented as percentages across all included studies.

**Figure 3.**
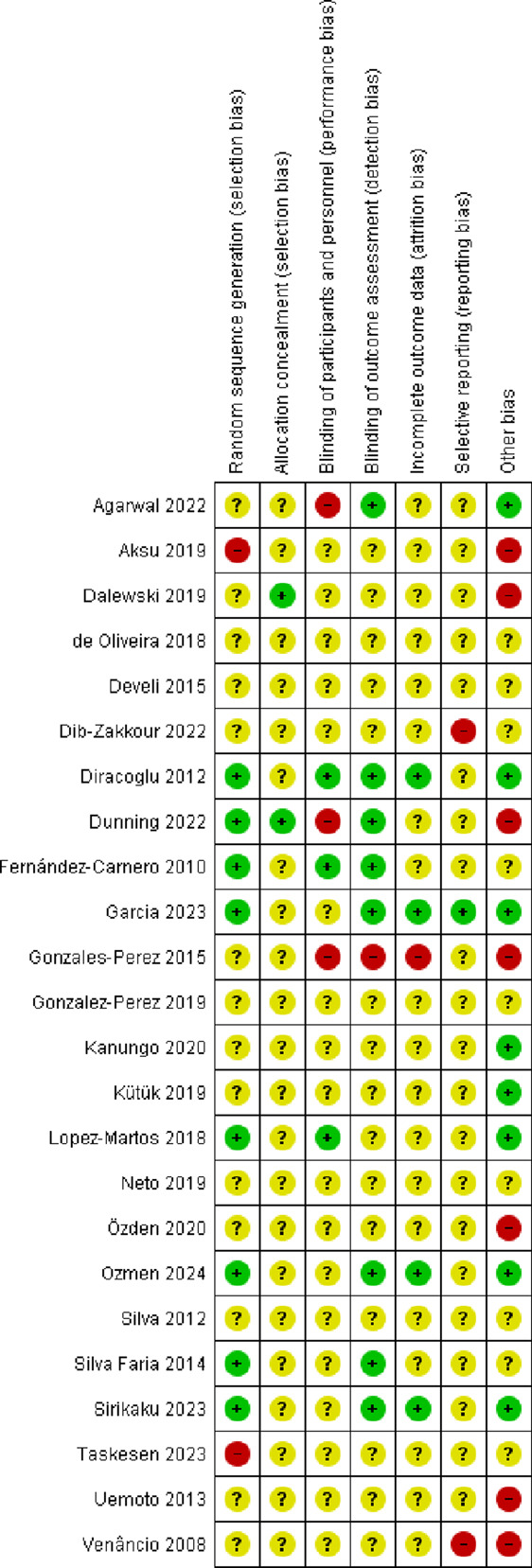
Risk of bias summary: review authors' judgements about each risk of bias item for each included study.

Our searches generated a total of 2,176 “hits.” After removal of duplicates, 1,338 titles and abstracts were screened for inclusion. Of those, 44 full-text articles were considered potentially relevant. A total of 24 RCTs with a total of 1,318 (mean sample size = 54.9) patients were eligible for inclusion (Fig. 1).^2,3,11–17,19–22,26,29,32,35,37,38,42–44,46,47^ Their key data are summarized in Supplementary Table 2 (available at http://links.lww.com/PR9/A260). Seventeen trials used DN as a standalone intervention^2,11,13–16,19–22,29,32,35,37,43,44,47^ and 7 in combination with other interventions, for example, lidocaine injections, usual care (UC), and exercise or muscular inhibition.^3,12,17,26,38,42,46^ Supplementary Table 3 (available at http://links.lww.com/PR9/A260) lists the AEs reported in RCTs. Supplementary Table 4 (available at http://links.lww.com/PR9/A260) presents details of the DN intervention. For the results of risk of bias assessments, see Figures 2 and 3.

### 3.2. Effectiveness of dry needling interventions

#### 3.2.1. Dry needling + usual care vs controls (usual care alone)

##### 3.2.1.1. Pain intensity (visual analogue scale)

**Figure 4.**
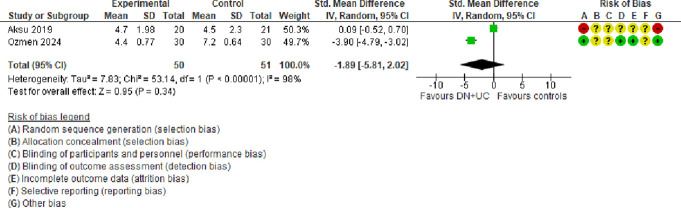
Forest plot of comparison: DN + UC vs controls (UC alone): outcome: pain intensity (VAS). CI, confidence interval; DN, dry needling; UC, usual care; VAS, visual analogue scale

A meta-analysis of 2 trials^3,38^ (1 judged to be at a very high risk of bias) showed that compared with UC alone, DN + UC had no effect on pain intensity (visual analogue scale) (SMD = −1.89, 95% CI −5.81 to 2.02, very low certainty evidence) at follow-ups of up to 6 weeks (Fig. 4). There was a considerable amount of heterogeneity (τ^2^ = 7.83; χ^2^ = 53.14, *I*^2^ = 98%) stemming from different interventions and UC protocols used.

#### 3.2.2. Dry needling vs sham (placebo)

##### 3.2.2.1. Facial pain

**Figure 5.**
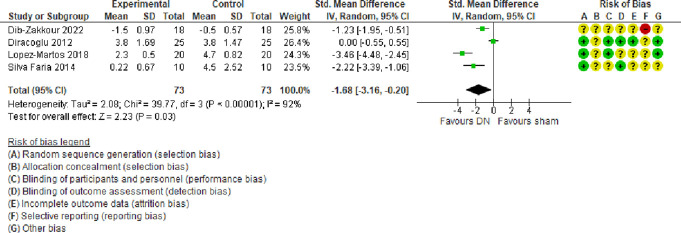
Forest plot of comparison: DN vs sham: outcome: facial pain. CI, confidence interval; DN, dry needling.

A meta-analysis of 4 trials^11,15,16,32^ (predominantly at a low or unclear risk of bias) showed that compared with sham, DN alone may reduce slightly facial pain intensity (SMD = −1.68, 95% −3.16 to −0.20, very low certainty evidence) at follow-ups ranging from 3 to 10 weeks (Fig. 5). There was a considerable amount of heterogeneity (τ^2^ = 2.08; χ^2^ = 39.77, *I*^2^ = 92%) stemming from the different magnitude and direction of effect sizes.

##### 3.2.2.2. Range of motion

**Figure 6.**
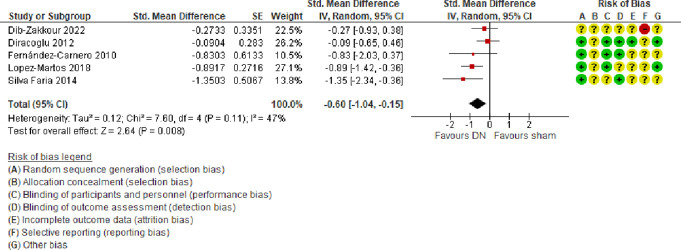
Forest plot of comparison: DN vs sham: outcome: ROM. CI, confidence interval; DN, dry needling; ROM, range of motion.

A meta-analysis of 5 trials^11,15,16,19,32^ (predominantly at a low or unclear risk of bias) showed that compared with sham, DN alone increased range of motion (SMD = −0.60, 95% CI −1.04 to −0.15, low certainty evidence) at follow-ups ranging from 3 to 10 weeks (Fig. 6). There was a moderate amount of heterogeneity (τ^2^ = 0.12; χ^2^ = 7.60, *I*^2^ = 47%).

##### 3.2.2.3. Pressure pain threshold

**Figure 7.**
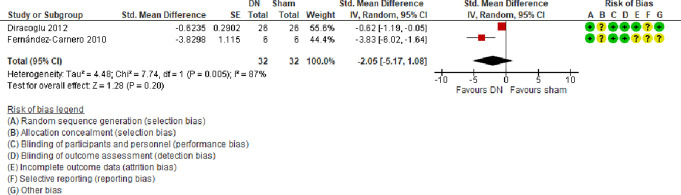
Forest plot of comparison: DN vs sham: outcome: pressure pain threshold (masseter). CI, confidence interval; DN, dry needling.

A meta-analysis of 2 trials^16,19^ (predominantly at an unclear risk of bias) showed that compared with sham, DN alone had no effect on pressure pain threshold at masseter (SMD = −2.05, 95% CI −5.17 to 1.08, very low certainty evidence) at 3-week follow-up (Fig. 7). There was a considerable amount of heterogeneity (τ^2^ = 4.48; χ^2^ = 7.74, *I*^2^ = 87%) stemming from different study designs used.

#### 3.2.3. Dry needling vs lidocaine injection

##### 3.2.3.1. Pain

**Figure 8.**
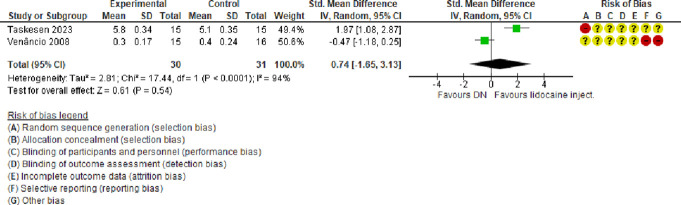
Forest plot of comparison: DN vs lidocaine injection: outcome: pain. CI, confidence interval; DN, dry needling.

A meta-analysis of 2 trials^44,47^ (both at a high or very high risk of bias) showed that compared with lidocaine injections, DN had no effect on pain (SMD = 0.74, 95% CI −1.65 to 3.13, very low certainty evidence) at 12-week follow-up (Fig. 8). There was a considerable amount of heterogeneity (τ^2^ = 2.81; χ^2^ = 17.44, *I*^2^ = 94%) stemming from different comparators used and the direction of effect sizes.

#### 3.2.4. Other comparators

For the remaining 8 comparisons with single studies, that is, DN + spinal manipulation therapy vs interocclusal splint therapy, nonsteroidal anti-inflammatory drugs and mobilization, DN vs manual therapy, DN vs UC, DN vs low laser, DN + lidocaine vs lidocaine alone, DN vs botulinum toxin, DN vs no intervention, and DN vs platelet rich plasma, please see the Appendix 2 (available at http://links.lww.com/PR9/A260).

### 3.3. Adverse effects

Only 6 RCTs (25%) mentioned AEs, and none of them reported that AEs had occurred.^3,17,22,32,37,38^ The remaining 18 (75%) studies failed to report AEs.

### 3.4. Risk of bias

Most RCTs had an unclear or high risk of bias, with baseline differences being the most common flaw in 7 studies (29.1%) (Figs. 2 and 3). Among 24 included trials, only 8 (33.3%) sufficiently explained an adequate randomisation process and were rated as being at a low risk of bias. Twenty-two studies (91.6%) employed an unclear method of sequence generation, meaning there was insufficient information to assign either high or low risk rating. We considered 2 studies (8.3%) to be at high risk of bias for random sequence generation.

### 3.5. Quality/certainty of the evidence (Grading of Recommendations Assessment, Development, and Evaluation)

The quality of the evidence ranged from very low to low for all comparisons/outcomes. Reasons for downgrading included inconsistency as substantial level of heterogeneity was detected in all but 1 meta-analyses: imprecision as trials were typically of small or very small sample sizes, and the CIs around the effects sizes were very wide; indirectness as there were differences in, for example, UC protocols (comparators); and study limitations (high risk of other bias).

## 4. Discussion

This SR was aimed at critically evaluating the available evidence for or against the effectiveness of DN for OFP. Twenty-four RCTs were included with a total of 1,318 participants. Overall, the results are contradictory and unconvincing.

None of the trials used the International Headache Society classification of OFP.^1^ This means clearly delineating idiopathic OFP from other pains, for example, resembling presentations of primary headaches or pain attributed to lesions of the cranial nerves was difficult. Also, it is worth noting that our SR included all types of OFP, whereas the other reviews have focused specifically on masticatory myofascial pain,^36,45^ OFP pain associated with temporomandibular disorder,^33,48^ and on myofascial pain in the craniofacial region.^27^

The description of the DN intervention was often suboptimal, making it difficult to replicate the studies. There was only 1 RCT assessing quality of life after DN interventions. Most of the included studies failed to report AEs associated with the DN interventions. This is a dereliction of research ethics and highlights generally poor quality of the research in this area. As a consequence, it is impossible to define the risks of DN or estimate its risk/benefit balance.

Using the Grading of Recommendations Assessment, Development, and Evaluation approach, the quality of the evidence was judged to be very low or low for all outcomes. Reasons for downgrading the evidence typically included inconsistency (considerable amount of statistical heterogeneity), study limitations (serious or very serious methodological flaws), imprecision (wide CIs around the effect estimates resulting from small or very small sample sizes), and indirectness (even UC protocols differed) (Supplementary Tables 5-7, available at http://links.lww.com/PR9/A260). Overall, there was a considerable indirectness of the evidence, that is, 11 (46%) different types of comparators were identified (almost every study using a dissimilar control and/or, discrepant treatment protocols). In 7 trials (29%), DN was used in combination with other interventions meaning possible confounding effects of cointerventions.^3,12,17,26,38,42,46^ However, the types of outcome measures used were relatively homogeneous, that is, pain, range of motion, and pressure pain threshold being the most common.

Several reviews (some unsystematic and, therefore, more susceptible to bias) evaluating DN in OFP have been published.^4,6,27,28,33,36,45,48^ Most of those reviews arrived at positive conclusions.^27,48^ We consider such positive conclusions to be unreliable for several reasons. The previous reviews included only a fraction of the studies that we managed to include in the present paper. They also failed to critically appraise the included evidence. Moreover, they pooled studies with heterogeneous interventions.

Strengths of this study include registration of the protocol, comprehensive searches, double-data extraction, critical appraisal of the literature, strict adherence to the Cochrane Handbook, Grading of Recommendations Assessment, Development, and Evaluation assessments, inclusion of unpublished trials, and statistical pooling. Our SR has several limitations, too. First, although our searches were comprehensive, we cannot guarantee that all relevant articles were located. Second, there was considerable amount of statistical heterogeneity for 3 outcomes, that is, *I*^2^ > 90% that stemmed from clinical and methodological differences in populations and interventions tested. Third, running subgroup analyses was not feasible due to the small number of studies under respective comparisons.

At present, DN cannot be considered as an effective treatment option for OFP. This is due to the uncertainties of the available evidence. We believe that larger, rigorous, and better reported trials with more homogeneous comparators might potentially reduce the current uncertainties. Such trials should strictly adhere to the classifications provided by the International Headache Society and published in the International Classification of Orofacial Pain.^1^

This SR generated disappointingly little compelling evidence for the effectiveness of DN in reducing OFP. The very low to low certainty of the evidence prevents firmly positive conclusions.

## Disclosures

All authors have no conflicts of interest to declare.

## Supplementary Material

SUPPLEMENTARY MATERIAL

## Supplemental digital content

Supplemental digital content associated with this article can be found online at http://links.lww.com/PR9/A260.
