# Supplementary material for: Dry needling for orofacial pain: a systematic review and meta-analysis of randomized clinical trials
Source: Pain Rep. 2024 Nov 20;9(6):e1208. doi: 10.1097/PR9.0000000000001208 (PMC11581761; doi:10.1097/PR9.0000000000001208)
Supplement: SUPPLEMENTARY MATERIAL [file painreports-9-e1208-s001.pdf]

Appendix 1: Search strategy for Medline

1. exp Face/
2. exp Facial Injuries/
3. (face or facial or nose$ or mouth$ or ear$ ADJ3 pain$).ti,ab.
4. Orofacial pain.tw.
5. exp craniomandibular disorders/
6. exp facial pain/
7. (temporomandibular or "temporo mandibular").mp.
8. (TMJ or TMD or TMJD).ti,ab.
9. ((TM or TMJ) adj (disorder$ or dysfunction$ or disease$)).mp.
10. ((facial or face or orofacial or "oro facial") adj2 (pain$ or neuralgia)).mp.
11. exp muscle tightness/
12. range of motion ADJ4 limitation$.ti,ab.
13. myofascial trigger points.tw.
14. exp muscle soreness/
15. exp myofascial pain/
16. exp musculoskeletal pain
17. #1 OR #2 OR #3 OR #4 OR #5 OR #6 OR #7 OR #8 OR #9 OR #10 OR #11 OR #12 OR #13 OR #14 OR #15 OR #16
18. Exp dry needling/
19. Exp analgesia/
20. dry needling.tw.
21. dry needling [MeSH Terms]
22. dry needl$ ADJ3 trigger point$.tw.
23. #18 OR #19 OR #20 OR #21 OR #22
24. 17 AND 23
25. randomized controlled trial.pt.
26. controlled clinical trial.pt.
27. randomi?ed.ab.
28. placebo.ab.
29. clinical trials as topic.sh.
30. randomly.ab.
31. trial.ti.
32. #24 OR #25 OR #26 OR #27 OR #28 OR #29 OR #30
33. exp animals/ NOT humans.sh.
34. #32 NOT #33
35. #24 AND #34

Appendix 2: Remaining comparisons with single studies

Supplementary figures 1-19.

	Experimental			Control			Std. Mean Difference	Std. Mean Difference
Study or Subgroup	Mean	SD	Total	Mean	SD	Total	IV, Random, 95% CI	IV, Random, 95% CI
de Oliveira 2018	35.2	3.56	5	36.2	6.46	5	-0.17 [-1.42, 1.07]	

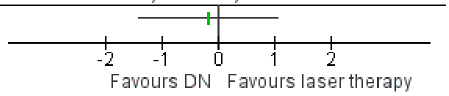

Forest plot of comparison: DN vs low laser: outcome: ROM (mouth opening)

	Experimental			Control			Std. Mean Difference	Std. Mean Difference
Study or Subgroup	Mean	SD	Total	Mean	SD	Total	IV, Random, 95% CI	IV, Random, 95% CI
Garcia 2023	9.44	3.08	25	8.12	3.79	25	0.38 [-0.18, 0.94]	

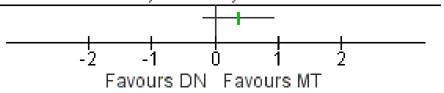

Forest plot of comparison: DN vs MT: outcome: disability

	Experimental			Control			Std. Mean Difference	Std. Mean Difference
Study or Subgroup	Mean	SD	Total	Mean	SD	Total	IV, Random, 95% CI	IV, Random, 95% CI
Garcia 2023	4.53	0.31	25	4.62	0.46	25	-0.23 [-0.78, 0.33]	

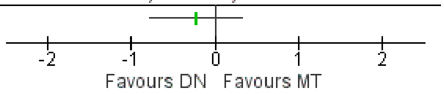

Forest plot of comparison: DN vs MT: outcome: ROM

	Experimental			Control			Std. Mean Difference	Std. Mean Difference
Study or Subgroup	Mean	SD	Total	Mean	SD	Total	IV, Random, 95% CI	IV, Random, 95% CI
Ozmen 2024	-33.7	4.75	30	-28.5	5.08	30	-1.04 [-1.59, -0.50]	

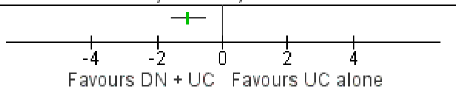

Forest plot of comparison: DN + UC vs UC alone: outcome: ROM (mouth opening)

	Experimental			Control				Std. Mean Difference	Std. Mean Difference
Study or Subgroup	Mean	SD	Total	Mean	SD	Total	Weight	IV, Random, 95% CI	IV, Random, 95% CI
Gonzales-Perez 2015	1.8	0.91	23	2	1.06	16	100.0%	-0.20 [-0.84, 0.44]	
**Total (95% CI)**			**23**			**16**	**100.0%**	**-0.20 [-0.84, 0.44]**	
Heterogeneity: Not applicable									
Test for overall effect: Z = 0.62 (P = 0.54)									

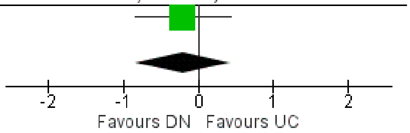

Forest plot of comparison: DN vs UC: outcome: pain

	Experimental			Control			Std. Mean Difference	Std. Mean Difference
Study or Subgroup	Mean	SD	Total	Mean	SD	Total	IV, Random, 95% CI	IV, Random, 95% CI
de Oliveira 2018	2.3	2.86	5	3.2	2.58	5	-0.30 [-1.55, 0.95]	

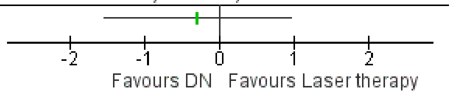

Forest plot of comparison: DN vs low laser: outcome: pain (VAS)

Study or Subgroup	Experimental Mean	SD	Total	Control Mean	SD	Total	Weight	Std. Mean Difference IV, Random, 95% CI
Kütük 2019	3.4	1.8	20	4.7	1.7	20	100.0%	-0.73 [-1.37, -0.09]
**Total (95% CI)**			**20**			**20**	**100.0%**	**-0.73 [-1.37, -0.09]**
Heterogeneity: Not applicable								
Test for overall effect: Z = 2.22 (P = 0.03)								

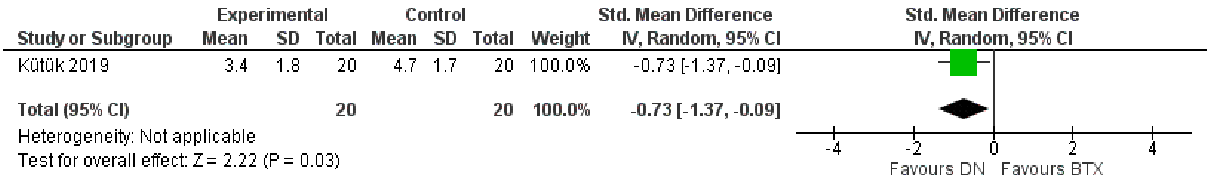

Forest plot of comparison: DN vs BTX: outcome: Pain at mastication

Study or Subgroup	Experimental Events	Total	Control Events	Total	Weight	Risk Ratio (Non-event) M-H, Fixed, 95% CI
Sirikaku 2023	11	20	9	20	100.0%	0.82 [0.44, 1.53]
**Total (95% CI)**		**20**		**20**	**100.0%**	**0.82 [0.44, 1.53]**
Total events	11		9			
Heterogeneity: Not applicable						
Test for overall effect: Z = 0.63 (P = 0.53)						

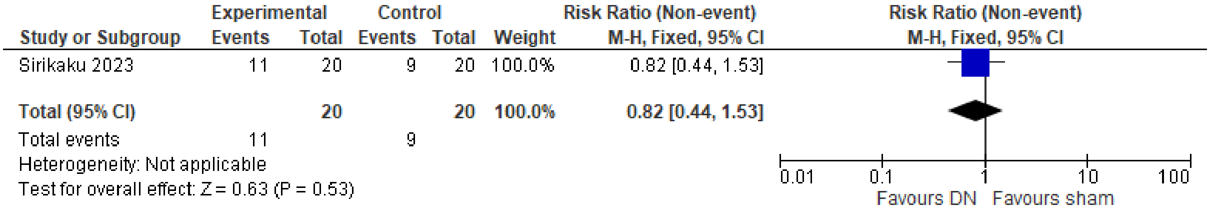

Forest plot of comparison: DN vs sham: outcome: improvement in pain (VAS)

Study or Subgroup	Experimental Mean	SD	Total	Control Mean	SD	Total	Std. Mean Difference IV, Random, 95% CI
Kanungo 2020	-83.3	12.58	100	-62.6	9.37	100	-1.86 [-2.19, -1.53]

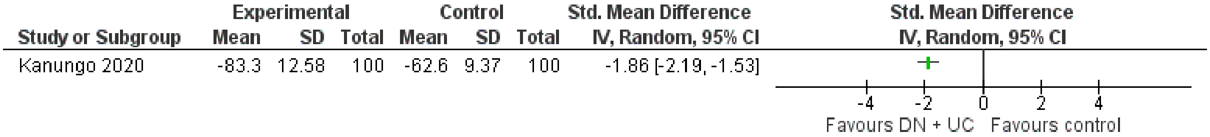

Forest plot of comparison: DN + UC vs UC alone: outcome: quality of life

Study or Subgroup	Experimental Mean	SD	Total	Control Mean	SD	Total	Std. Mean Difference IV, Random, 95% CI
Garcia 2023	3.48	2	25	2.84	1.95	25	0.32 [-0.24, 0.88]

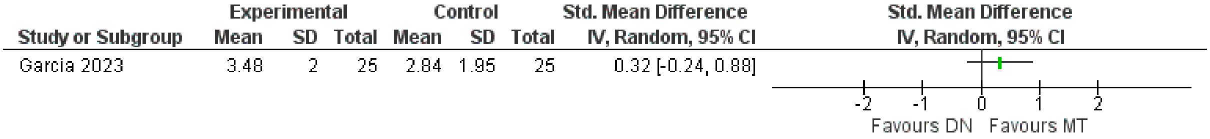

Forest plot of comparison: DN vs MT: outcome: pain (NPRS)

Study or Subgroup	Experimental Mean	SD	Total	Control Mean	SD	Total	Weight	Std. Mean Difference IV, Random, 95% CI
Kütük 2019	-45	5.8	20	-43.7	5	20	100.0%	-0.24 [-0.86, 0.39]
**Total (95% CI)**			**20**			**20**	**100.0%**	**-0.24 [-0.86, 0.39]**
Heterogeneity: Not applicable								
Test for overall effect: Z = 0.74 (P = 0.46)								

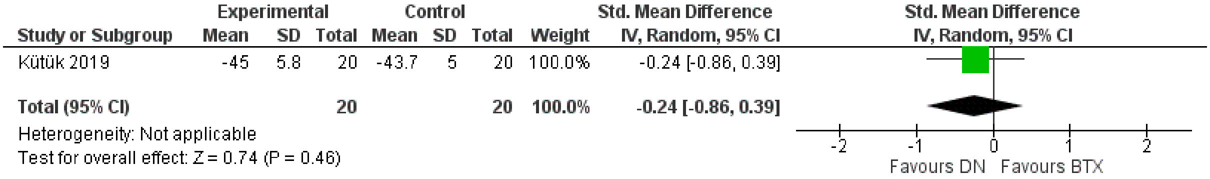

Forest plot of comparison: DN vs BTX: outcome: ROM (mouth opening)

Study or Subgroup	Experimental Mean	SD	Total	Control Mean	SD	Total	Std. Mean Difference IV, Random, 95% CI
Dunning 2022	-44.1	7.9	62	-35.4	7.4	58	-1.13 [-1.51, -0.74]

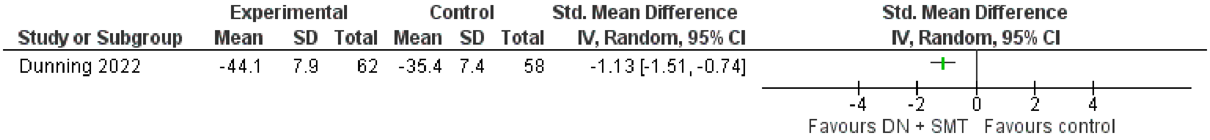

Forest plot of comparison: DN + SMT vs control: outcome: ROM (mouth opening)

Study or Subgroup	Experimental Mean	SD	Total	Control Mean	SD	Total	Weight	Std. Mean Difference IV, Random, 95% CI
Kütük 2019	3.1	1.8	20	4.1	1.4	20	100.0%	-0.61 [-1.24, 0.03]
**Total (95% CI)**			**20**			**20**	**100.0%**	**-0.61 [-1.24, 0.03]**
Heterogeneity: Not applicable								
Test for overall effect: Z = 1.87 (P = 0.06)								

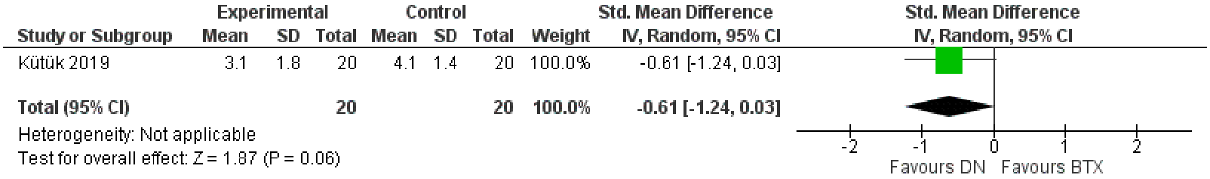

Forest plot of comparison: DN vs BTX: outcome: Pain at rest

Study or Subgroup	Std. Mean Difference	SE	Weight	Std. Mean Difference IV, Random, 95% CI	Std. Mean Difference IV, Random, 95% CI
Gonzales-Perez 2015	-0.386	0.3288	100.0%	-0.39 [-1.03, 0.26]	
**Total (95% CI)**			**100.0%**	**-0.39 [-1.03, 0.26]**	
Heterogeneity: Not applicable					
Test for overall effect: Z = 1.17 (P = 0.24)					

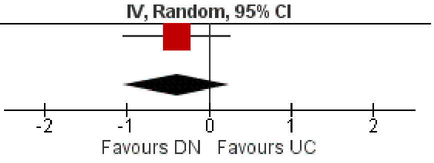

Forest plot of comparison: DN vs UC: outcome: pain at mastication

Study or Subgroup	Experimental Mean	SD	Total	Control Mean	SD	Total	Weight	Std. Mean Difference IV, Random, 95% CI	Std. Mean Difference IV, Random, 95% CI
Taskesen 2023	37.6	0.74	15	37.8	1.37	15	100.0%	-0.18 [-0.89, 0.54]	
**Total (95% CI)**			**15**			**15**	**100.0%**	**-0.18 [-0.89, 0.54]**	
Heterogeneity: Not applicable									
Test for overall effect: Z = 0.48 (P = 0.63)									

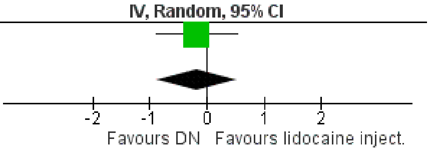

Forest plot of comparison: DN vs lidocaine injection: outcome: Max mouth opening (ROM)

Study or Subgroup	Std. Mean Difference	SE	Weight	Std. Mean Difference IV, Random, 95% CI	Std. Mean Difference IV, Random, 95% CI
Gonzales-Perez 2015	-0.9146	0.3499	100.0%	-0.91 [-1.60, -0.23]	
**Total (95% CI)**			**100.0%**	**-0.91 [-1.60, -0.23]**	
Heterogeneity: Not applicable					
Test for overall effect: Z = 2.61 (P = 0.009)					

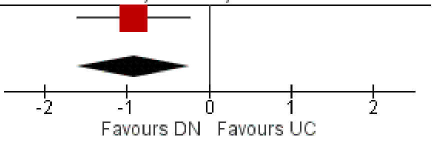

Forest plot of comparison: DN vs UC: outcome: ROM

Study or Subgroup	Experimental Mean	SD	Total	Control Mean	SD	Total	Weight	Std. Mean Difference IV, Random, 95% CI	Std. Mean Difference IV, Random, 95% CI
Agarwal 2022	42.4	8.36	15	44.6	6.69	15	100.0%	-0.28 [-1.00, 0.44]	
**Total (95% CI)**			**15**			**15**	**100.0%**	**-0.28 [-1.00, 0.44]**	
Heterogeneity: Not applicable									
Test for overall effect: Z = 0.77 (P = 0.44)									

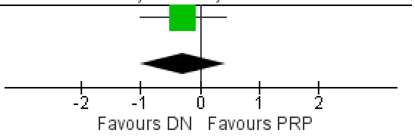

Forest plot of comparison: DN vs platelet rich plasma: outcome: ROM mouth opening

Study or Subgroup	Experimental Mean	SD	Total	Control Mean	SD	Total	Weight	Std. Mean Difference IV, Random, 95% CI	Std. Mean Difference IV, Random, 95% CI
Lopez-Martos 2018	3.2	0.9	20	4.2	1.49	20	100.0%	-0.80 [-1.44, -0.15]	
**Total (95% CI)**			**20**			**20**	**100.0%**	**-0.80 [-1.44, -0.15]**	
Heterogeneity: Not applicable									
Test for overall effect: Z = 2.41 (P = 0.02)									

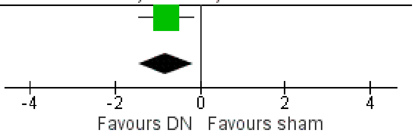

Forest plot of comparison: DN vs sham: outcome: pain at mastication

Study or Subgroup	Experimental Mean	SD	Total	Control Mean	SD	Total	Weight	Std. Mean Difference IV, Random, 95% CI	Std. Mean Difference IV, Random, 95% CI
Agarwal 2022	2	1.96	15	0.6	1.05	15	100.0%	0.87 [0.11, 1.62]	
**Total (95% CI)**			**15**			**15**	**100.0%**	**0.87 [0.11, 1.62]**	
Heterogeneity: Not applicable									
Test for overall effect: Z = 2.25 (P = 0.02)									

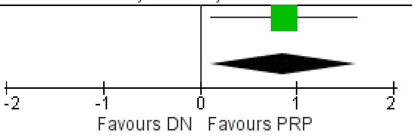

Forest plot of comparison: DN vs platelet rich plasma: outcome: pain (VAS)

Supplementary Tables: 1-7

Table 1: Eligibility criteria

Item	Included	Excluded
**Population**	OFP (chronic or acute)*	Rhinosinusitis, migraine, cervicogenic or cluster headache
**Intervention**	Dry needling (DN)** (as a standalone intervention or in combination) delivered by physical therapists or physiotherapists	e.g., acupuncture, acupressure, electroacupuncture, bee venom, homeopuncture
**Comparator**	Any	-
**Outcome**	• Efficacy (pain severity/ intensity, duration, function, range of motion<br>• safety (adverse effects)<br>• health related quality of life	-
**Study design**	Randomised controlled trials***	Non-randomised studies; observational studies
**Timeframe**	No limits	-
* = regardless of the classification used<br>** = also includes neuromodulation and electro DN<br>*** = including split face, cross-over		

Table 2: Randomized controlled studies of DN for the treatment of OFP

First author (Year) [reference]	Study design	Participants (n)	IHS criteria for OFP (yes/no)	Experimental intervention (details see Tab 3)	Control intervention	Primary outcome measure	Main result	Comment
Agarwal (2022)[1]	Parallel RCT with 2 groups	30	No	DN	Platelet-rich plasma	1. Pain (VAS)<br>2. ROM	1. SMD = 0.87 [95% CI 0.11, 1.62]* at 6 months<br>2. SMD = -0.28 [-1.00, 0.44]* at 3 months	Pain on function favoured controls
Aksu, (2019)[2]	Quasi RCT with 3 groups	63	No	1. DN + exercise + protection training	1. Trigger point injection + exercise + protection training<br>2. exercise + protection training	1. Pain (VAS)<br>2. Pain (mouth opening)<br>3. Functional limitation<br>4. Tender points of facial and neck	1. SMD = 0.09 [-0.52, 0.70; (P = 0.557)]* at one month<br>2. N.s.<br>3. N.s.<br>4. N.s.	Within group differences reported; small sample; confounding effects of exercise and protection training

						muscles (algometry)		
Dalewski (2019)[10]	Parallel RCT with 3 groups	90	No	1. DN + occlusal appliance	1. NSAID + occlusal appliance<br>2. Occlusal appliance alone	1. Pain (VAS)	1. n.s.	No power and sample size calculations; significant difference for NSAID vs occlusal appliance alone
Develi (2015)[12]	Parallel RCT with 4 groups	100	No	DN	1. Lidocaine injection<br>2. Sham DN<br>3. BTX-A injection	1. Pain (VAS)<br>2. Pressure pain<br>threshold	1. n.s.<br>2. n.s.	Abstract only (limited information)
Dib-Zakkour (2022)[13]	Parallel RCT with 2 groups	36	No	DN	Sham DN	1. Pain (VAS)<br>2. Pressure pain<br>threshold<br>3. ROM mouth opening (pattern)	1. SMD = -1.23 [95% CI -1.95, -0.51]*<br>2. n.r.<br>3. SMD = -0.27 [95% CI -0.93, 0.38]*	Poor reporting; no baseline characteristics
Diracoglu (2012)[14]	Double blind RCT	52	No	DN	Sham DN	1. Pain (VAS)	1. SMD = 0.00 [95% CI	Relatively well-designed; low drop-out rate

	with 2 parallel groups					2. Pressure pain threshold<br>3. ROM mouth opening (pattern)	-0.55, 0.55]* at 3 weeks<br>2. SMD = -0.62 [95% CI -1.19, -0.05]* at 3 weeks<br>3. SMD = -0.09 [95% CI -0.65, 0.46]* at 3 weeks	
Dunning (2022)[15]	Parallel RCT with 2 groups	120	No	DN+ Upper Cervical SMT	Interocclusal Splint Therapy, NSAIDs and Mobilization	1. jaw pain intensity (VAS)<br>2. ROM -pain-free mouth opening (mm)	1. MD = −21.9 [95% CI −29.1, −14.7; (P< 0.001)] at 3 months<br>2. MD = 9.1 [95% CI 7.1, 11.1]; (P< 0.001)] at 3 months	Baseline differences in age, gender and duration of symptoms

Fernández-Carnero (2010)[17]	Cross-over RCT with 2 groups	12	No	DN	Sham DN	1. Pressure pain threshold<br>a. masseter<br>b. mandibular condyle<br>2. ROM mouth opening	1. a. SMD= -3.83 [95% CI -6.02, -1.64; (P = 0.0006)]*<br>1. b. SMD = -3.64 [95% CI -5.75, -1.53; (P = 0.0007)]*<br>2. SMD = -0.83 [95% CI -2.03, 0.37; P = 0.18]*	High possibility of contamination of the outcomes with carry-over effects; very small sample
Garcia (2023)[18]	Parallel RCT with 2 groups	50	No	DN	MT (neuromuscular technique)	1. Pain (NPRS)<br>2. Disability<br>3. ROM	1. SMD = 0.32 [95% CI -0.24, 0.88]*<br>2. SMD = 0.38 [95% CI -0.18, 0.94]*<br>3. SMD = -0.23 [95% CI -0.78, 0.33]*	Pre-registered and well-designed trial
Gonzalez-Perez (2015) [20]	Open RCT	48	No	DN	UC (drug therapy)	1. a. Pain (VAS) at rest	1. a. SMD = -0.20 [95%	No power calculations; small sample,

	with 2 groups					b. at mastication<br>2. ROM (mouth opening)	CI -0.84, 0.44]*<br>1. b. SMD = -0.39 [95% CI -1.03, 0.26]*<br>2. SMD = -0.91 [95% CI -1.60, -0.23]*	33% lost to follow-up in the UC arm; lack of blinding
Gonzalez-Perez (2019)[19]	Parallel RCT with 3 groups	45	No	DN	1. Intratissue<br>percutaneous electrolysis<br>2. BTX-A injection	1.Pain<br>a. at rest<br>b. at mastication<br>2. maximum interincisal opening	1. a. sig.<br>b. sig.<br>2. sig. (all for DN v BTX-A at 16 weeks)	Abstract only (limited information)
Kanungo (2020)[24]	Parallel RCT with 2 groups	200	No	DN + muscular inhibition in combination with home based exercise	Home based exercise	QoL (SF-36)	SMD = -1.86 [95% CI -2.19, -1.53]* for bodily pain	Poor reporting; relatively large sample size
Kütük (2019)[27]	Parallel RCT	40	No	DN	BTX-A injection	1. Pain (VAS)<br>a. at rest	1. a. SMD = -0.61 [95% CI -1.24,	Small sample; imprecision

	with 2 groups					b. at mastication<br>2. ROM (mouth opening)	0.03]* at 6 weeks<br>b. -0.73 [95% CI -1.37, -0.09]* at 6 weeks<br>2. SMD = -0.24 [-0.86, 0.39]* at 6 weeks	
Lopez-Martos (2018)[29]	Double-blind RCT with 3 parallel groups	60	No	DN	1. Intratissue<br>percutaneous electrolysis<br>2. Sham	1. a. Pain at rest<br>b. at mastication<br>2. ROM-maximum interincisal opening	1. a. SMD = -3.46 [95% CI -4.48, -2.45]* at day 70<br>b. SMD =-0.80 [95% CI -1.44, -0.15]* at day 70<br>2. SMD = -0.89 [95% CI -1.42, -0.36]* at day 70	Small sample; no power calculations

Neto (2019)[32]	Quasi RCT with 2 parallel groups	30	No	DN	MT	1. Pain (VAS)<br>2. Pressure pain threshold	1. n.s.<br>2. n.s.	Abstract only (limited information)
de Oliveira (2018)[11]	Parallel RCT with 2 groups	10	No	DN	Low intensity laser therapy	1. ROM - mouth opening<br>2. Pain (VAS)	1. SMD = -0.17 [95% CI -1.42, 1.07]* at 7 weeks<br>2. SMD = -0.30 [95% CI -1.55, 0.95]* at 7 weeks	Very small sample and imprecise findings
Özden (2020)[34]	Parallel RCT with 3 groups	60	No	Deep DN	1. Superficial DN<br>2. No intervention	1. Pain (VAS)<br>2. Pressure pain threshold<br>3. ROM - mouth opening	1-3. inestimable	Only within group differences; means and SDs not reported
Ozmen (2024)[35]	Parallel RCT with 3 groups	90	No	DN+UC	1. Face yoga+UC<br>2. UC+soft food diet, heat application	1. Pain (VAS)<br>2. ROM - mouth opening	1. SMD = -3.90 [95% CI -4.79, -3.02]* at 6 weeks<br>2. SMD = -1.04 [95% CI	Relatively (to other trials) large sample; and well reported

							-1.59, -0.50]* at 6 weeks	
Silva (2012) [39]	Parallel RCT with 2 groups	16	No	DN + lidocaine hydrochloride 0.5% injection	DN only	1. Pain (VAS)<br>2. Pressure pain threshold	1. inestimable<br>2. SMD = -0.73 [95% CI -1.75, 0.29]* at 30 days	Very imprecise findings; poor reporting
Silva Faria (2014) [9]	Single-blind RCT with 3 parallel groups	30	No	DN	1. Sham<br>2. Counselling	1. Pain (VAS)<br>2. ROM - (unassisted jaw opening without pain)	1. SMD = -2.22 [95% CI -3.39, -1.06]* at 3 weeks<br>2. SMD =<br>-1.35 [95% CI -2.34, -0.36]* at 3 weeks	Imprecise findings; no power calculations; only age at baseline reported
Sirikaku (2023)[40]	Single-blind RCT with 2	25	No	DN	Sham	Pain (VAS)	RR = 0.82 [0.44, 1.53]* at 3 months	Small sample; imprecise findings; blinding of personnel was not ensured

	parallel groups							
Taskesen (2023) [41]	Quasi RCT with 3 groups	45	No	DN	1. Masseteric nerve block<br>2. Trigger point injection (lidocaine)	1. Pain on palpation<br>2. Pain on function (VAS)<br>3. Pain free mouth opening (ROM)	1. n.s. (P= 0.765)<br>2. SMD = 1.97 [95% CI 1.08, 2.87]* at 12 weeks<br>3. SMD = -0.18 [95% CI -0.89, 0.54]	Pain on function favoured controls (lidocaine); underpowered
Uemoto (2013)[43]	Parallel RCT with 3 groups	21	No	DN + lidocaine	1. Laser therapy<br>2. Control (placebo)	1. Pain (VAS)<br>2. ROM (mouth opening)<br>3 . Pressure pain threshold	1. n.s.<br>2. n.s. (P = 0.1563)<br>3. n.s.	Mainly within group differences (poorly) reported; very small sample; no baseline data provided
Venâncio Rde (2008) [44]	Parallel RCT with 3 groups	45	No	DN	1. lidocaine<br>2. lidocaine + corticosteroid	1. Symptom Severity Index (pain)<br>2. Pain (palpation)<br>3. Pain diary	1. SMD = -0.47 [95% CI -1.18, 0.25]* at 12 weeks<br>2. inestimable<br>3. n.r.	Pain diary and pain questionnaire not reported

						4. Pain questionnaire	4. n.r.	
	BTX = botulinum toxin; CI = confidence interval; DN = dry needling; IHS = International Headache Society; MT = manual therapy; MD = mean difference; NPRS = Numeric Pain Rating Scale; NSAID = nonsteroidal anti-inflammatory drug; n.s. = No significant between-group differences at post intervention (unless otherwise specified); OFP = Orofacial Pain; QoL=quality of life; ROM = range of motion; SD = standard deviation; SF-26 = Short Form-36 questionnaire; SMD = standardized mean difference; SMT = spinal manipulative therapy; UC = usual care; VAS= visual analogue scale.<br>* = calculated with RevMan 5.4							

Table 3: Adverse effects (AEs) reported in RCTs

Study (year)	Details of adverse effects reported
Agarwal (2022)[1]	n.r.
Aksu, (2019)[2]	None reported
Dalewski (2019)[10]	n.r.
Develi (2015)[12]	n.r.
Dib-Zakkour (2022)[13]	n.r.
Dunning (2022)[15]	"No major adverse events were reported in the dry needling and upper cervical spinal manipulation group."
Diracoglu (2012) [14]	n.r.
Garcia-de la-Banda-Garcia (2023)[18]	n.r.
Gonzalez-Perez (2015) [20]	"No adverse reactions were detected with DDN, whereas up to 41% of the patients receiving the combination drug treatment described unpleasant side effects (drowsiness)."

Gonzalez-Perez (2019)[19]	n.r.
Fernández-Carnero 2010[17]	n.r.
Kanungo (2020)[24]	n.r.
Kütük (2019)[27]	n.r.
Lopez-Martos (2018)[29]	"No adverse reactions were detected with DN"
Neto (2019)[32]	n.r.
de Oliveira (2018)[11]	n.r.
Özden (2020)[34]	None reported
Ozmen (2024)[35]	"In this study, no adverse effects were encountered in the dry needling group."
Taskesen (2023) [41]	n.r.
Silva (2012) [39]	n.r.
Silva Faria (2014) [9]	n.r.
Sirikaku (2023)[40]	n.r.
Uemoto (2013)[43]	n.r.

Venâncio Rde (2008) [44]	n.r.
n.r = not reported; UC = usual care	

Table 4: Details of the DN intervention

Study (year)	Details of the DN intervention (direct quote where applicable)
Agarwal (2022)[1]	"DN was performed in the TrPs in the masseter muscle with a 1.5-inch, 27-gauge needle but no solution"
Aksu, (2019)[2]	"The trigger point in the right or left or bilateral masseter and lateral pterygoid muscles was detected by palpation. An acupuncture needle was applied to the point and the needle was turned around itself once in five min. The needle was kept in the muscle for 20 min until the muscle became relaxed."
Dalewski (2019)[10]	"Three visits were needed for this [DN] treatment modality. Visits schedule: first visit-day 1, second visit-7 days after the first, and third visit-7 days after the second. Equipment: acupuncture needle of dimensions 0.6 ×13mm (Dragon Medical Device Ltd., China), solution for disinfection of skin (Octenisept, Schulke and Mayr GmbH), and sterile gauze 5 × 5 cm (Mato, Poland). Exposition time: 30 minutes once a week."
Develi (2015)[12]	"and dry needling [was delivered] to each TrP in the last group, followed by stretching of the muscle groups involved"

Dib-Zakkour (2022)[13]	“Group E received the DN treatment in both masseter muscles, using 0.30 x 0.30 mm AGUPUNT acupuncture needles with guides. The technique requires the patient to rest in a supine position, with their eyes closed and with the head rotated towards the right when treating the left masseter muscle and towards the left for the right masseter muscle”
Dunning (2022)[15]	“All participants received up to eight treatment sessions at a frequency of once or twice per week over a 4-week period. In either group, fewer treatment sessions could be completed if symptom resolution occurred sooner. […] Patients allocated to the experimental group received up to eight sessions of DN at a frequency of 1–2 times per week for 4 weeks using a standardized protocol of 7 points for 20 min, as described in Appendix 1.”
Diracoglu (2012) [14]	“ […] DN with intramuscular stimulation was applied on the trigger points using standard single-use sterile acupuncture needles (0.22 mm × 30 mm) with plastic guide tubes (3 times with 7-day intervals). The needle was inserted to the depth allowed by the guide tube and was stimulated 3 or 5 times”
Garcia-de la-Banda-Garcia (2023)[18]	„This [DN] treatment consisted of puncturing the possible active MTrP of the right or left masseter, lateral pterygoid, and sternocleidomastoid muscles. Patients in this group received dry needling in at least 1 active trigger point and in a maximum of 6. Sterile needles, 0.26 mm in diameter by 40 mm in length, guided with

	a plastic cannula of the brand Ener-qi, were used. The area was cleaned with alcohol and deep puncturing of the myofascial trigger point was performed, triggering local spasm responses. The needle was moved up and down through the muscle following the technique described by Hong. After the procedure, the area was compressed with cotton for 90 s.”
Gonzalez-Perez (2015) [20]	“The DDN group received needling of the lateral pterygoid muscle once per week for 3 weeks. […] For the DDN therapy, sterile stainless steel needles (length 40 mm/ caliber 0.25 mm, with a cylindrical plastic guide; Agu-punt ®) were used. […] Intramuscular needling was then carried out. This was performed via a deep puncture into myofascial pain TPs without the introduction of any substance (dry needling)”
Gonzalez-Perez (2019)[19]	“The DN group received a deep puncture to the TrP without the introduction of any substance.”
Fernández-Carnero 2010[17]	“Each participant attended two treatment sessions at least 7 days apart and received one intervention […] at the most painful point on the masseter muscle. Both interventions were administered by a therapist with more than 5 years of clinical experience in dry needling. For both interventions, needles used for this experiment were stainless steel, manufactured by Novasan (Maraca “Ener-Qi” CE0197).

	The needle size used for the study was different for each intervention: in the experimental (deep dry needling) condition, an acupuncture needle (0.26 x 25 mm) was used, whereas in the sham intervention a shorter needle was employed (0.26 x 13 mm)"
Kanungo (2020)[24]	"[…] dry needling was performed on masseter, temporalis and sub-occipital muscle for first weeks for three session the in the second week muscle inhibition technique were performed for medial and lateral ptregoid, masseter, temporalis, as well as for sub-occipital muscle. Before using the dry needling the skin surfaces was cleaned by saline water the a plastic guided 40mm acupuncture needle was insert in to the tender point of the muscle and for muscle inhibition technique , ischemic sustained pressure applied on the muscle for 5-10 second."
Kütük (2019)[27]	" […] in the DN treatment, 38mm long needle with a green tip was used. Following the appropriate skin preparation, the needle was held perpendicular to the skin and quickly the subcutaneous tissue was entered. The needle was inserted into the muscle until the trigger point in the muscle band with the tip was found. The same point was needled rapidly 8 to 10 times with the tip of the needle mounted to the empty syringe"

Lopez-Martos (2018)[29]	"A deep intramuscular puncture of the TPs was carried out without the introduction of any substance (dry puncture). […- During the procedure, the operator used the volume of the electrotherapy equipment as a guide, simulating the EPI® technique"
Neto (2019)[32]	n.r.
de Oliveira (2018)[11]	"patients were provided with 6 sessions of DN, unilaterally in 2 patients and bilaterally in 8 patients, according to the complaint of where the patient was experiencing pain and the presence of TP. DN was made with sterile acupuncture needles (DongBang Acupuncture®, Boryeong, Chungnam, Korea) with a 0.25 x 30mm caliber and 5cm long enveloped by a cylindrical plastic holder 4.5cm long. After the needle insertion, smooth and rotating movements were performed for 1 minute in each TP".
Özden (2020)[34]	"Sterile stainless-steel needles with a plastic cylindrical guide, 25 mm in length and 0.25 mm in diameter, were used for superficial DN. A total of three sessions were performed per patient per week, and clinical assessments were performed at three and six weeks after finishing the treatment. The needle was applied after asepsis of the area with 90% alcohol and manual location of the masseter, with an intramuscular needling depth of up to 5 mm. […]"

Ozmen (2024)[35]	"Sterile stainless steel needles (Hualong Dry Needle, Shanghai, China) with a length of 13 mm and a diameter of 0.25 mm, equipped with a plastic cylindrical guide, were utilized for dry needling. Prior to the procedures, the skin was cleaned with an appropriate antiseptic. Dry needling was performed by inserting the needles into the masseter and temporal muscles, starting just below the zygomatic arch and entering the mandibular angle region 2.5 cm in front of the tragus. Immediately after insertion, the needle was rotated clockwise and left in place for 10 min, reversed once again after 10 min. Each dry needling session lasted for a total of 20 min. Dry needling sessions were conducted three times a week for a duration of 6 weeks."
esen (2023) [41]	"In the DN group, needling therapy was performed using disposable sterile acupuncture needles. The initial insertion depth of the needle was adjusted by the guide tubes. Rapid needling with winding was performed three to five times. […] DNs were performed two times with a 7-day interval."
Silva (2012) [39]	No details provided (for the DN group).
Silva Faria (2014) [9]	"DN therapy was applied in the MTrPs using standard single use sterile acupuncture needles 0,20mm x 13mm. Each patient received 3 sessions with 7 days intervals. The patient was placed in the supine

	position, the skin was disinfected with alcohol, the trigger points were determined and the needle was inserted. When the needle penetrated the MTrP a movement “up and down” was repeated 3 to 5 times (without being completely removed). The procedure was repeated for several MTrPs (active and latent).”
Sirikaku (2023)[40]	“Sterile stainless-steel needles with dimensions of 13 mm long and 0.20 mm diameter were used (Origin: China, manufacturer: Wujiang), individually packed and accompanied by a cylindrical plastic slider. Trigger point location was secured, immobilized between the index and middle fingers of the non-dominant hand. Then, the cylindrical slider was positioned with a slight pressure on the skin to produce a tactile stimulus in order to minimize the discomfort caused by the insertion of the needle. The DN procedure was based on the needle method described by Hong and the needle was inserted perpendicular to the masseter muscle, through the skin to the trigger point, and then, a slow insertion and partial withdrawal of the needle were performed, and at each insertion […]”
Uemoto (2013)[43]	“ […] DN of MTPs located in the right masseter muscle. The same muscle on the left side was injected with 0.25 ml of 2% lidocaine without epinephrine (Lidostesim SV-Dentsply brand, São Paulo, Brazil). Dental carpules with reflux and short 30G (Unoject Nova DFL brand, Rio de Janeiro, Brazil) disposable

	needles were utilized. The needle was inserted to a depth of 1 to 2 cm at an acute angle of 30° to the skin, in various directions, with movement into the tissue.”
Venâncio Rde (2008) [44]	“Once a trigger point has been located and the overlying skin has been cleansed with alcohol, the clinician isolates this point with by pinching it between his/her fingers and then inserts the needle 1-2 cm away from the trigger point, so that the needle may be advanced into the trigger point at an acute angle of 30 degrees to the skin”
DN = dry needling; MTrP/MTP = myofascial trigger points; n.r. = not reported	

Table 5: Summary of findings

DN compared to sham for OFP

Patient or population: Patients with OFP

Setting: Clinics

Intervention: DN

Comparison: sham

Outcomes	Anticipated absolute effects* (95% CI)		Relative effect (95% CI)	№ of participants (studies)	Certainty of the evidence (GRADE)	Comments
	**Risk with sham**	**Risk with DN**				
Pressure Pain Threshold - Masseter	-	SMD **2.05 lower** (5.17 lower to 1.08 higher)	-	(2 studies)	⊕◯◯◯ Very low[c,d,e]	
Pressure Pain Threshold - Lateral epicondyle	-	SMD **3.64 lower** (5.75 lower to 1.53 lower)	-	(1 study)	⊕◯◯◯ Very low[f,g,h]	Fernández-Carnero (2010) (N = 12) reported that compared with sham, DN may increase pressure pain threshold at mandibular condyle (very low certainty)

DN compared to sham for OFP

Patient or population: Patients with OFP

Setting: Clinics

Intervention: DN

Comparison: sham

Outcomes	**Anticipated absolute effects**[*] (95% CI)		Relative effect (95% CI)	№ of participants (studies)	Certainty of the evidence (GRADE)	Comments
	**Risk with sham**	**Risk with DN**				
ROM (mouth opening)	-	SMD **0.6 lower** (1.04 lower to 0.15 lower)	-	190 (5 RCTs)	⨁⨁◯◯ Low[a,b]	
Facial pain (VAS)	-	SMD **1.68 lower** (3.16 lower to 0.2 lower)	-	146 (4 RCTs)	⨁◯◯◯ Very low[a,c,d]	

DN compared to sham for OFP

Patient or population: Patients with OFP

Setting: Clinics

Intervention: DN

Comparison: sham

Outcomes	**Anticipated absolute effects*** (95% CI)		Relative effect (95% CI)	№ of participants (studies)	Certainty of the evidence (GRADE)	Comments
	**Risk with sham**	**Risk with DN**				
Pain at mastication	-	SMD **0.8 lower** (1.44 lower to 0.15 lower)	-	(1 study)	⊕◯◯◯ Very low[d,g,h]	Lopez-Martos (2018) (N = 60) reported that compared with sham, DN may reduce slightly pain at mastication at day 70 (very low certainty)
Improvement in pain (VAS) categorical	450 per 1,000	**369 per 1,000** (198 to 689)	**RR 0.82** (0.44 to 1.53)	(1 study)	⊕⊕◯◯ Low[d,g]	Sirikaku (2023) (N = 25) reported that compared with sham, DN has no effect on pain at 3 months (low certainty)

***The risk in the intervention group** (and its 95% confidence interval) is based on the assumed risk in the comparison group and the **relative effect** of the intervention (and its 95% CI).

CI: confidence interval; DN: dry needling; OFP: orofacial pain; QoL: quality of life; ROM: range of motion; RR: risk ratio; SMD: standardised mean difference; UC: usual care; VAS: visual analogue scale.

**DN compared to sham for OFP**
**Patient or population:** Patients with OFP<br>**Setting:** Clinics<br>**Intervention:** DN<br>**Comparison:** sham

Outcomes	**Anticipated absolute effects*** (95% CI)		Relative effect (95% CI)	№ of participants (studies)	Certainty of the evidence (GRADE)	Comments
	**Risk with sham**	**Risk with DN**				

GRADE Working Group grades of evidence
Low certainty: our confidence in the effect estimate is limited: the true effect may be substantially different from the estimate of the effect.
Very low certainty: we have very little confidence in the effect estimate: the true effect is likely to be substantially different from the estimate of effect.

Explanations

a. Downgraded for studies limitations: Dib-Zakkour 2022 judged to be at a high risk of bias (-1).

b. I^2 = 47% heterogeneity (-1).

c. Downgraded for inconsistency as there was a considerable amount of heterogeneity (-1).

d. Downgraded for imprecision: small sample size and wide confidence intervals around effect estimate (-1).

e. Downgraded for studies limitations: both RCTs were judged to be at an overall unclear risk of bias (-1).

f. Downgraded for study limitations: judged to be at an overall unclear risk of bias (-1).

g. Downgraded as results were obtained from a single study (−1).

h. Downgraded for imprecision: very small sample size (-2).

Table 6: Summary of findings

DN compared to lidocaine injection for OFP

Patient or population: Patients with OFP

Setting: Various clinics

Intervention: DN

Comparison: lidocaine injection

	Anticipated absolute effects* (95% CI)					
**Outcomes**	**Risk with lidocaine injection**	**Risk with DN**	**Relative effect (95% CI)**	**№ of participants (studies)**	**Certainty of the evidence (GRADE)**	**Comments**
Pain (measured with: Symptom Severity Index)	-	SMD **0.74 higher** (1.65 lower to 3.13 higher)	-	61 (2 RCTs)	⊕◯◯◯ Very low[a,b,c]	-
Max mouth opening (ROM)	-	SMD **0.18 lower** (0.89 lower to 0.54 higher)	-	30 (1 study)	⊕⊕◯◯ Low[d,e]	Taskesen (2023) (N = 45) reported that compared with lidocaine injection, DN may have no effect on maximum mouth opening (low certainty)

Table 6: Summary of findings

**DN compared to lidocaine injection for OFP**						
**Patient or population:** Patients with OFP<br>**Setting:** Various clinics<br>**Intervention:** DN<br>**Comparison:** lidocaine injection						
**Outcomes**	**Anticipated absolute effects* (95% CI)**		**Relative effect (95% CI)**	**№ of participants (studies)**	**Certainty of the evidence (GRADE)**	**Comments**
	**Risk with lidocaine injection**	**Risk with DN**				

***The risk in the intervention group** (and its 95% confidence interval) is based on the assumed risk in the comparison group and the **relative effect** of the intervention (and its 95% CI).
CI: confidence interval; DN: dry needling; OFP: orofacial pain; ROM: range of motion; SMD: standardised mean difference.

GRADE Working Group grades of evidence
Low certainty: our confidence in the effect estimate is limited: the true effect may be substantially different from the estimate of the effect.
Very low certainty: we have very little confidence in the effect estimate: the true effect is likely to be substantially different from the estimate of effect.

Explanations

a. Downgraded as both studies (Taskesen 2023 and Venâncio 2008) at a high or very high risk of bias (−2).

b. Downgraded for inconsistency as there was a considerable heterogeneity: I^2 = 94% (-2).

c. Downgraded for imprecision as small sample size; and wide confidence intervals around effect estimate (-1).

d. Downgraded as study (Taskesen 2023) was judged to be a high risk of bias (−1).

e. Downgraded as results were obtained from a single study (−1).

Table 7: Summary of findings

**DN + UC compared to controls (UC alone) for OFP**						
**Patient or population:** Orofacial pain<br>**Setting:** Clinics<br>**Intervention:** DN + UC<br>**Comparison:** Controls (UC alone)						
**Outcomes**	**Anticipated absolute effects* (95% CI)**		**Relative effect (95% CI)**	**№ of participants (studies)**	**Certainty of the evidence (GRADE)**	**Comments**
	**Risk with controls (UC alone)**	**Risk with DN + UC**				
Pain (VAS)	-	SMD **1.89 lower** (5.81 lower to 2.02 higher)	-	101 (2 RCTs)	⨁◯◯◯ Very low[a,b,c,d]	-
QoL	-	SMD **1.86 lower** (2.19 lower to 1.53 lower)	-	200 (1 study)	⨁⨁◯◯ Low[e,f]	Kanungo (2020) (N = 200) reported that compared with UC alone, DN + UC may improve QoL (low certainty)
ROM (mouth opening)	-	SMD **1.04 lower** (1.59 lower to 0.5 lower)	-	60 (1 study)	⨁⨁◯◯ Low[d,f]	Ozmen (2024) (N=90) reported that compared with UC alone, DN + UC may improve ROM (low certainty)

**DN + UC compared to controls (UC alone) for OFP**

Patient or population: Orofacial pain

Setting: Clinics

Intervention: DN + UC

Comparison: Controls (UC alone)

Outcomes	Anticipated absolute effects* (95% CI)		Relative effect (95% CI)	№ of participants (studies)	Certainty of the evidence (GRADE)	Comments
	Risk with controls (UC alone)	Risk with DN + UC				

*__The risk in the intervention group__ (and its 95% confidence interval) is based on the assumed risk in the comparison group and the **relative effect** of the intervention (and its 95% CI).
CI: confidence interval; DN: dry needling; OFP: orofacial pain; QoL: quality of life; ROM: range of motion; SMD: standardised mean difference; UC: usual care; VAS: visual analogue scale.

GRADE Working Group grades of evidence

Low certainty: our confidence in the effect estimate is limited: the true effect may be substantially different from the estimate of the effect.
Very low certainty: we have very little confidence in the effect estimate: the true effect is likely to be substantially different from the estimate of effect.

Explanations

a. Downgraded as Aksu (2019) judged to be at a very high risk of bias (−1).

b. Downgraded for inconsistency as there was a 98% heterogeneity (−1).

c. Downgraded for indirectness as even usual care protocols differed (−1).

d. Downgraded for imprecision; small sample size (−1).

e. Downgraded as study was judged to be an unclear risk of bias (−1).

f. Downgraded as results were obtained from a single study (−1).
